# Coding rules for uncertain and “ruled out” diagnoses in ICD-10 and ICD-11

**DOI:** 10.1186/s12911-024-02661-6

**Published:** 2024-09-27

**Authors:** Oluseun O. Atolagbe, Patrick S. Romano, Danielle A. Southern, Wachira Wongtanasarasin, William A. Ghali

**Affiliations:** 1 Health Information Management Department, University of California Davis Health, Sacramento, CA USA; 2 Departments of Internal Medicine and Pediatrics, and Center for Healthcare Policy and Research, University of California Davis Health, Sacramento, CA USA; 3 Centre for Health Informatics, Cumming School of Medicine, University of Calgary, Calgary, AB Canada; 4 Department of Emergency Medicine, Faculty of Medicine, Chiang Mai University, Chiang Mai, Thailand; 5 Office of Vice President of Research, University of Calgary, Calgary, AB Canada

**Keywords:** Classification, International Classification of Diseases, ICD-11, Uncertain diagnoses, Ruled out

## Abstract

The International Classification of Diseases, 11th Revision (ICD-11) has significantly improved the ability to navigate coding challenges beyond prior iterations of the ICD. Commonly encountered sources of complexity in clinical documentation include coding of uncertain and “ruled out” diagnoses. Assessing official international guidelines and rules, this paper documents extensive variation across countries in existing practices for coding and reporting unconfirmed and “ruled out” clinical concepts in ICD-10 (and modifications thereof). The design of ICD-11 is intended to mitigate these coding challenges by introducing postcoordination, expanding the range of codable clinical concepts, and offering clearer guidance in the ICD-11 Reference Guide. ICD-11 offers substantial progress towards more precise capture of uncertain and “ruled out” diagnoses, including international consensus on coding rules for these historically challenging clinical concepts. However, we identify the need for further clarification of the concepts of “provisional diagnosis” and “differential diagnosis.”

## Background

The International Statistical Classification of Diseases and Related Health Problems (also known as the International Classification of Diseases, ICD) has evolved over several generations into a classification system for capturing healthcare mortality and morbidity data, thus permitting comparisons of health care services and outcomes across organizations, communities, and countries [1]. Although ICD originated as a tool for compiling international mortality statistics, the classification system has evolved significantly, and is currently being used to code clinical documentation and classify morbidity for resource allocation, budgeting, case-mix, patient safety and quality metrics, primary care, and research [2, 3]. With this broader set of ICD use cases, the 11th Revision of ICD (ICD-11) involved focused work on enhancing the Classification’s structure, content, and ease of use, while capturing more modern and specific clinical concepts, consistent with World Health Organization (WHO) goals.

There are many instances in clinical medicine where the primary focus of an encounter is to rule out certain conditions, or where uncertainty about the patient’s correct diagnoses remains at the end of the encounter. Recognizing this fact, it is desirable for a modern classification system to have features that provide unambiguous coding guidance for such situations. Variation in relevant coding guidance within and across countries limits the value of ICD-10 coded data for surveillance and epidemiology, clinical and health services research, provider payment and budgeting, and quality and safety use cases [4]. Harmonization of ICD coding standards and guidelines was recently identified as a priority by classification experts from 26 countries [5]. An international survey of 246 users of coded health data by the WHO Quality and Safety Topic Advisory Group, in preparation for efforts to enhance ICD-11 [6], highlighted this issue as well as the inability to link clinical concepts by clustering codes.

Practical steps have been taken in the design of ICD-11 to mitigate challenges in coding uncertain or unconfirmed diagnoses. ICD-11 has powerful new features that unlock mechanisms for capturing uncertain or unconfirmed diagnoses, including postcoordination (or clustering) of relevant concepts and improved tooling to support more consistent coding practices. In this paper, we review the current situation with ICD-10 and explore related opportunities and challenges presented by ICD-11.

## Methods

To evaluate historical and proposed coding rules with respect to uncertain and “ruled out” diagnoses, we undertook a careful review of current practices in ICD-10 and all English-language modifications thereof, including:

- The International Classification of Diseases, 10th Revision, Clinical Modification (ICD-10-CM), which is used in the United States and United Arab Emirates.
- The International Statistical Classification of Diseases and Related Health Problems, 10th Revision, Canada (ICD-10-CA), which is used across Canada [7].
- The International Statistical Classification of Diseases and Related Health Problems, 10th Revision, Australian Modification (ICD-10-AM), which is used in Australia, New Zealand, Ireland, Saudi Arabia, Ukraine, and several other Eastern European, south Pacific, and Gulf countries [8–10].
- The International Statistical Classification of Diseases and Related Health Problems, Tenth Revision, Thai Modification (ICD-10-TM), which is used in Thailand [11].

We then explored how the design of ICD-11 is intended to improve reporting of uncertain and “ruled out” diagnoses, with specific attention to the value of postcoordination and the potential need for additional clarity in WHO’s instructions to health care practitioners and coders.

### Reporting uncertain diagnoses in ICD-10

Clinical circumstances often arise where diagnoses are considered during care but may still be unconfirmed at the end of the episode of care. These unconfirmed or uncertain diagnoses are sometimes described as “rule out” diagnoses, and they are typically documented in the medical record using qualifiers such as “probable,” “suspected…,” “likely…,” “?”, “questionable…,” “possible…,” “consider…,” “ruling out…(R/O),” “consistent with… (c/w),” and other terms indicating ambiguity [12, 13]. The ICD-10 Instruction Manual’s guidance on *morbidity* coding states that diagnoses qualified as “possible,” “questionable,” or “suspected” should be coded to “the greatest degree of specificity and knowledge of the condition that necessitated care or investigation… by stating a symptom, abnormal finding or problem” [14]. However, WHO created one limited exception for the main condition, defined in ICD-10 “as the condition, diagnosed at the end of the episode of health care, primarily responsible for the patient’s need for treatment or investigation.” If “after an episode of health care, the main condition is still recorded as ‘suspected’, ‘questionable’, etc., and there is no further information or clarification, the suspected diagnosis must be coded as if established.” For example, “suspected acute cholecystitis” is coded as “acute cholecystitis (K81.0)” only if it qualifies as the main condition.

South African ICD-10 Coding Standards do not recognize the WHO’s exception for the main condition [15]. In South Africa, any “diagnosis recorded as “possible” or “suggestive of” or “probable” or prefixed with a “?” or “query” will not be coded as if the given diagnosis is confirmed regardless of the treatment that has been provided to the patient.” In such circumstances, only the relevant symptoms are coded because qualifying terms of uncertainty are “taken to mean that there remained a significant element of doubt as to the actual diagnosis and that the differential diagnoses were still being considered (or that the patient appeared to be recovering so further investigations were not being undertaken but that there was a significant level of uncertainty over the actual diagnosis).” Similarly, the Standard Coding Guidelines for ICD-10-TM from the Thai Ministry of Public Health instruct users, “a common mistake is to diagnose ‘rule out (R/O)’… this term should not be used since it implies that a clinician cannot make a definite diagnosis or needs further investigations.” Clinicians and coders are instructed to code the sign or symptom “rather than giving the diagnosis ‘R/O disease’,” even when the clinician is awaiting or cannot obtain diagnostic test results, or when the patient is transferred to another hospital or leaves the hospital before testing is complete [11].

In the United States’ implementation of ICD-10, the ICD-10 Clinical Modification (ICD-10-CM), Official Guidelines for Coding and Reporting [12] state that ANY condition documented at the time of discharge from a short-term, acute, long-term care, or psychiatric hospital “as ‘probable,’ ‘suspected,’ ‘likely,’ ‘questionable,’ ‘possible, or ‘still to be ruled out,’ ‘compatible with,’ ‘consistent with,’ or other similar terms indicating uncertainty” should be coded “as if it existed or was established” (regardless whether or not it is the “principal diagnosis,” in US terminology). The basis for this instruction is “the diagnostic workup, arrangements for further workup or observation, and initial therapeutic approach that correspond most closely with the established diagnosis.” In other words, these Official Guidelines ensure that if a patient was treated in a manner consistent with a specific diagnosis, but a definitive test was not performed or is not yet available, then the facility can be paid (using Medicare Severity Diagnosis Related Groups or other diagnosis-based classifiers) as if the diagnosis was established. However, for outpatient services, uncertain or “rule out” diagnoses are not coded. Rather, in accord with the WHO’s ICD-10 Instruction Manual [14], these conditions are coded “to the highest degree of certainty for that encounter/visit, such as symptoms, signs, abnormal test results, or other reason for the visit.”

The Australian Coding Standards (ACS) for the Australian Modification of ICD-10, ICD-10-AM, include instructions similar to the United States, with an interesting variation. Standard 0012 distinguishes the *number* of suspected but uncertain diagnoses and advises coders that if “a single condition is suspected, assign a code for the suspected condition.” For example, “the patient was discharged with a diagnosis of ‘?lower respiratory tract infection (LRTI).’ Code: J22 Unspecified acute lower respiratory infection.” However, if “more than one suspected condition is documented as the differential diagnosis, assign code(s) for the documented symptom(s) OR if there are no symptom(s) documented, assign codes for all suspected conditions” [16]. For example, coders are instructed to assign R06.0 (dyspnoea) and R06.2 (wheezing) for a “patient admitted with shortness of breath and wheezing… discharged with a diagnosis of ‘?asthma ?bronchiectasis.’” Australian standards make no distinction among “terms that indicate uncertainty about the final diagnosis (such as probable, suspected, possible, likely, query, ?) or other similar qualifying expressions” [16], such as “differential dx” [17]. To accommodate the particular scenario of patients transferred between facilities with a suspected condition, ICD-10-AM adds Z75.6, “transfer for suspected condition,” to be “sequenced directly after the diagnosis code to which it relates” [18].

The Canadian Coding Standards (CCS) for the International Statistical Classification of Diseases and Related Health Problems, Tenth Revision, Canada (ICD-10-CA) [7] take a different approach to coding conditions documented with terms denoting uncertainty, including “query,” “suspected,” “questionable,” “rule out,” “possible,” “probable,” “likely”, “?” “presumed,” and “versus.” When a single unconfirmed diagnosis is recorded as the final diagnosis and there is no further information or clarification, Canadian coders are instructed to “assign a code for the unconfirmed diagnosis as if it were established, with a prefix “Q” denoting provider-documented uncertainty. For example, if “the final diagnosis is recorded by the physician as “query peptic ulcer,” code “(Q) K27.9 Peptic ulcer, unspecified as acute or chronic, without hemorrhage or perforation.” When two (or more) unconfirmed diagnoses are recorded with no further information or clarification, the first-listed unconfirmed diagnosis is reported as the main problem, with the prefix Q. Neonatal sepsis is an exception to this coding standard, in that the CCS requires clarification of documentation of “rule out sepsis” by the provider before coding. An unconfirmed diagnosis of neonatal sepsis cannot be reported, as it is clinically implausible that a neonate would be discharged (except by transfer to another hospital) without confirming the presence or absence of sepsis. Canadian Coding Standards regarding uncertain diagnoses apply in the same manner to data submitted to the Discharge Abstract Database as well as the National Ambulatory Care Reporting System, in contrast with the United States ICD-10-CM Official Guidelines, which differ for inpatient and outpatient reporting.

In its National Clinical Coding Standards ICD-10 5th Edition, the United Kingdom’s National Health Service [19] has taken a middle ground between the WHO’s ICD-10 Instruction Manual and the United States’ ICD-10-CM Official Guidelines, advising coders that “it is not always possible for the responsible consultant to provide a definitive (confirmed) diagnosis in the medical record… but they may be treating or investigating the patient’s condition based on a ‘presumed’ or ‘probable’ diagnosis.” Coders are asked to seek the advice of the responsible consultant for clarification, as in other international instructions, but “if it is not possible to get advice from the responsible consultant,” coders are authorized to report any diagnosis recorded as being treated or investigated, based on terms “recorded in the medical record (such as) ‘working diagnosis,’ ‘treat as’, ‘presumed’ or ‘probable’.” By contrast, diagnoses described in the context of “a differential diagnosis whilst working to determine which one of several diseases may be producing the symptoms in the absence of any further information” (using terms such “‘likely’ or ‘likelihood’”) are not reported. Rather, the main symptoms must be coded, as in these examples:

- “*Probable* Myocardial infarction: I21.9 Acute myocardial infarction, unspecified”
- “Abdominal pain – *likely* appendicitis: R10.4 Other and unspecified abdominal pain.”

### Reporting “ruled out” diagnoses in ICD-10

A related clinical concept is the “ruled out” diagnosis, which includes circumstances in which a suspected disease or condition was excluded following investigation during the encounter. ICD-10 contains a dedicated section, Z03, “Medical observation and evaluation for suspected diseases and conditions, *ruled out*” in Chapter 21– Factors influencing health status and contact with health services, which are reported for persons presenting with “symptoms or evidence of an abnormal condition which requires study, but who, after examination and observation, show no need for further therapeutic or medical care.” Under this definition, Z03 codes apply when there is certainty at the end of the encounter that the patient did not have the originally suspected diagnosis, even when their actual diagnosis remains uncertain. For example [14]:

- “Main condition: Admitted for investigation of suspected malignant neoplasm of cervix – ruled out. Code to: Observation for suspected malignant neoplasm (Z03.1) as ‘main condition’.
- Main condition: Ruled out myocardial infarction. Code to: Observation for suspected myocardial infarction (Z03.4) as ‘main condition’.”

The ICD-10-CM Official Guidelines in the United States generally discourage coding of conditions that have been ruled out, as the only conditions that qualify for coding are those “that coexist at the time of admission, that develop subsequently, or that affect the treatment received and/or the length of stay. Diagnoses that… have no bearing on the current hospital stay are to be excluded.” Thus, Z03 “ruled out” codes are allowed “in very limited circumstances”; for example, codes from Z03.7, “encounter for suspected maternal and fetal conditions ruled out” may be used when an encounter is for a suspected maternal or fetal condition that is ruled out during that encounter (for example, a maternal or fetal condition may be suspected due to an abnormal test result)… these codes are not for use if an illness or any signs or symptoms related to the suspected condition or problem are present… In such cases the diagnosis/symptom code is used” [12, 13]. Accordingly, ICD-10-CM omits many of WHO’s Z03 codes that the United States does *not* consider as appropriate for coding and reporting, including:

- Z03.0, Observation for suspected tuberculosis
- Z03.1, Observation for suspected malignant neoplasm
- Z03.2, Observation for suspected mental and behavioural disorders
- Z03.3, Observation for suspected nervous system disorder
- Z03.4, Observation for suspected myocardial infarction
- Z03.5, Observation for other suspected cardiovascular diseases
- Z03.9, Observation for suspected disease or condition, unspecified

However, ICD-10-CM adds a set of codes for conditions “ruled out” at Z04.7, “encounter for examination and observation following alleged physical abuse,” and Z05, “encounter for observation and evaluation of newborn for suspected diseases and conditions ruled out.” Note that the adjective “alleged” at Z04.4 (ICD-10) for rape, and at Z04.7 (1CD-10-CM only) for physical abuse, implies that a criminal act may or may not have been ruled out.

Most other English-speaking countries also discourage or limit use of Z03 observation codes for “ruled out” conditions. For example, Canadian Coding Standards for ICD-10-CA suggest assigning a code from category Z03 only when “the suspected condition is ruled out/not found; and there is no documentation to support that further investigation is required; and another underlying condition is not identified” [7]. In its National Clinical Coding Standards ICD-10 5th Edition, the United Kingdom’s National Health Service [19] also limits the use of Z03 codes. For example, in the exact scenario above where WHO recommends use of Z03.4 (“observation for suspected myocardial infarction”), British coders are advised to code R07.4 (“Chest pain, unspecified”), followed by I24.9 (“Acute ischaemic heart disease, unspecified”) if appropriate. Similarly, South African ICD-10 Coding Standards [15] advise that “when a sign and/or symptom is the reason for the examination, assign an appropriate code for the sign and/or symptom when no abnormality is detected.” Australian Coding Standards limit the use of Z03 codes to asymptomatic conditions, as “if symptoms related to the suspected condition are noted, then the symptom codes are assigned, not Z03-” [16].

As these examples illustrate, the lack of clustering or postcoordination mechanisms for linking these Chapter 21 (Z) codes with clinical concepts located in other chapters has led to international variation in coding guidelines and efforts to discourage reporting of these Chapter 21 codes in most English-speaking countries.

### ICD-11 Coding rules for “rule-out” diagnoses

It is often desirable to report unconfirmed diagnoses that necessitate complex diagnostic procedures or therapeutic interventions during an encounter, especially when the results of those procedures or interventions are not available when the record must be coded. In other instances, conditions qualified as “ruled out” need to be reported, by virtue of investigations, evaluations, resource use, and often, treatment provided during the inpatient stay. ICD-11 is better able to capture these conditions by linking clinical concepts through postcoordination, modifying and expanding relevant chapters, and revising applicable coding standards.

In ICD-11, a clinical condition may be captured by combining (linking or clustering) multiple codes to provide additional details, using a mechanism of ‘postcoordination’. Postcoordination is a notable new feature in ICD-11 that offers the ability to combine two or more stem codes with a front slash (i.e., stem code1/stem code2); or link stem codes with one or multiple extension codes, using an ampersand sign (i.e., stem code1 & extension code1 & extension code2) [2]. Detailed description of ‘postcoordination’ and code clustering is provided elsewhere in this Supplement [20]. Extension codes can be classified as type 1 codes, which describe the severity, temporality, anatomy, histopathology, or other dimensions of a stem code, and type 2 codes, which “indicate how the diagnosis is to be used and/or interpreted”; for example, the concept of “diagnosis certainty” (i.e., “XY7Z Provisional diagnosis” or “XY75 Differential diagnosis”) [2].

Just as in ICD-10, the WHO’s ICD-11 Reference Guide at 2.23.9 instructs that a suspected diagnosis must be coded as if established, “if, after an episode of health care, the *main* condition is recorded as ‘suspected’, ‘questionable’, etc., and there is no further information or clarification” [21]. For example:

- “Main condition: Suspected acute cholecystitis. If there is no further information available that indicates that a definitive diagnosis was reached, code to acute cholecystitis, unspecified (DC12.0Z) as ‘main condition’.
- Main condition: Severe epistaxis. Patient in hospital one day. No procedures or investigations reported. Code to epistaxis (MD20). Although epistaxis is a sign/symptom, it is acceptable since the patient was obviously admitted to deal with the immediate emergency only.”

To support coders in identifying codable conditions, the WHO’s ICD-11 Reference Guide also instructs health care practitioners that “if no definite diagnosis has been established at the end of an episode of health care, then the health care practitioner should document the information that permits the greatest degree of specificity and knowledge about the reason for admission that has been established at the end of the episode of care [22]. This could be a symptom, abnormal finding, or problem. Rather than qualifying a diagnosis as “possible”, or “suspected”, when a diagnosis has been considered but not established, when applicable, record the symptom, abnormal finding, or problem.” By following these instructions, health care providers will limit the frequency of instances in which “the *main* condition is recorded as ‘suspected’, ‘questionable’, etc.” However, the ICD-11 Reference Guide is silent on how to code uncertain diagnoses that qualify for coding but are not the main condition, and it does not define the terms “provisional diagnosis” and “differential diagnosis.”

**Fig. 1:**
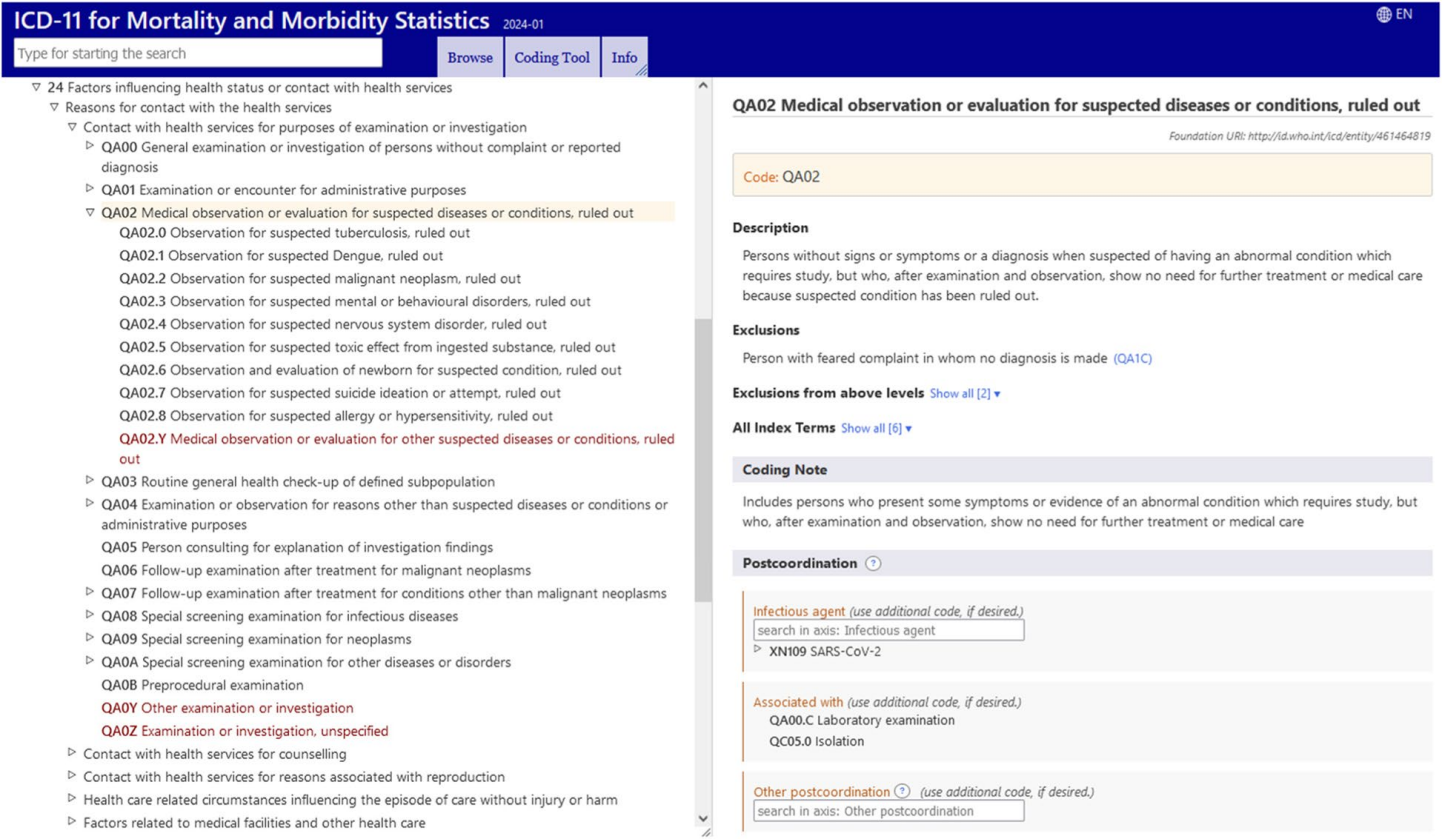
Chapter 24 block of codes for medical observation or evaluation for suspected diseases or conditions, *ruled out*. Source: https://icd.who.int/browse/2024-01/mms/en#461464819; accessed 11 June 2024

Circumstances in which suspected diagnoses are “ruled out” prior to discharge can also be captured in ICD-11, either as stand-alone codes or in a postcoordinated manner using codes from Chapter 24, Factors influencing health status or contact with health services, under “QA02 Medical observation or evaluation for suspected diseases or conditions, *ruled out*.” Fig. 1. These codes specify the condition of interest as “ruled out”, such as QA02.2, “observation for suspected malignant neoplasm, *ruled out*.” In cases where the ruled-out condition is not listed under the QA02 block of codes, QA02.Y Medical observation or evaluation for other suspected diseases or conditions, *ruled out* may be postcoordinated with “a code from another chapter to add specificity as to what was the suspected disease that was ruled out.” For example:

- Patient is admitted for suspected tuberculosis, which is *ruled out* after extensive study. Code: QA02.0 Observation for suspected tuberculosis, *ruled out*.
- Admission for suspected deep vein thrombosis of right leg, which is *ruled *out, after investigation. Code: QA02.Y/BD71.4 & XK9K (QA02.Y Medical observation or evaluation for other suspected diseases or conditions, *ruled out*; BD71.4 Lower limb deep vein thrombosis; Laterality: Right leg)

In the first example, a single code is available in Chapter 24, which precisely captures the clinical concept of suspected tuberculosis, ruled out. The second example requires additional information to capture the clinical concept to the desired level of detail, because the QA02 block of codes does not contain a code for the suspected diagnosis, deep vein thrombosis. QA02.Y indicates that a medical condition was ruled out and ICD-11’s postcoordination feature allows additional specification of the ruled-out condition (i.e., deep vein thrombosis of right leg). By convention, the ICD-11 Reference Guide dictates that QA02.Y should be sequenced *first* in such code clusters.

## Discussion

The new structure of the ICD-11 presents numerous opportunities for more flexibility and more pragmatic approaches to coding. As described above, the introduction of postcoordination and code clustering allows users to identify any condition as a “provisional diagnosis” (XY7Z), as part of a “differential diagnosis” (XY75), or as pursuant to medical observation or evaluation for a suspected disease or condition, ruled out (QA02.Y). As a result, users can take advantage of individual concepts, or combinations of concepts, to report conditions to desired levels of detail. Ultimately, complete and precise capture of diagnostic concepts in ICD-11 should improve the integrity and value of administrative data for various use cases.

Despite opportunities for improvement in coding of uncertain and “ruled out” diagnoses, there are potential challenges to intended use of these codes. Complete and accurate code reporting depend largely on the quality of provider documentation, as well as the knowledge and proficiency of the coder about the clinical concepts that are documented. Consequently, adequate training for health care providers and coders regarding coding guidelines and appropriate documentation of uncertain diagnoses would improve overall quality of coded information. Further, depending on the envisioned use case for code reporting, such as hospital payment or quality measurement, coding professionals may underreport uncertain diagnoses that do not impact payment. The ICD-11 Reference Guide could provide clearer instructions about how to code uncertain diagnoses that are not the main condition, and about when to use postcoordinated codes for “diagnosis certainty” (XY7Z or XY75). Clarity is also needed around whether ICD-11 should retain the ICD-10 convention (in most country-specific guidelines) of coding “ruled out” diagnoses *only* when further investigation is *not* required, and another underlying condition is *not* identified. For example, if a patient is admitted with chest pain and dyspnea with a suspected diagnosis of pulmonary embolus, but that diagnosis is ruled out by appropriate imaging, and the patient is later established to have acute pericarditis, should only pericarditis be coded or should the ruled-out diagnosis of pulmonary embolus also be coded with an appropriate stem code (QA02.Y)?

Health care providers use various terms for uncertain diagnoses that may be confusing for coders. Commonly used terms, such as “probable,” “suspected,” “likely,” “questionable,” “presumed,” and “ruling out,” are familiar. However, some symbols and even more ambiguous terms are found in medical records; for example, “?”, “compatible with,” “consistent with,” “r/o,” and “A versus B.” In such instances, coding professionals should be encouraged to confer with the health care provider for clarification of documentation, prior to code assignment, as is currently done in many healthcare organizations. The British distinction between “presumed” and “probable” diagnoses, which are coded as if they exist, and “likely” diagnoses, which are not, seems inexplicable and unlikely to generalize to other English-speaking countries. However, for uniformity of reporting, it may help to update the WHO ICD-11 Reference Guide with additional terms for easier reference, as was done in the United States (i.e., the terms “compatible with” and “consistent with” were added in October 2019). Terms suggesting especially low probabilities of a diagnosis, such as “consider,” “possible,” “differential,” and “unlikely,” could be recommended for postcoordination with “XY75 Differential diagnosis” instead of “XY7Z Provisional diagnosis.”

## Conclusions

More precise coding of uncertain and “ruled out” conditions in health information systems is of value to health care organizations, national health information systems, and researchers, particularly if there is interest in why and how much resources are being expended. Unambiguous reporting of these diagnoses, without invoking the ICD-10-CM practice of coding uncertain conditions as if they exist, may be useful for better understanding resource use and for assessing the impact of such health care encounters on health systems. The refinement of coding rules and concepts for uncertain and “ruled out” diagnoses is another important new feature of ICD-11 that positions the new classification to capture richer and better data on health encounters.

## Data Availability

Not applicable.

## Abbreviations

ICD: International Classification of Diseases
ICD-11: International Classification of Diseases, 11th revision
ICD-10: International Classification of Diseases, 10th revision
ICD-10-AM: International Statistical Classification of Diseases and Related Health Problems, 10th Revision, Australian Modification
ICD-10-CM: International Classification of Diseases, 10th Revision, Clinical Modification
ICD-10-CA: International Statistical Classification of Diseases and Related Health Problems, 10th Revision, Canada
CCS: Canadian Coding Standards
WHO: World Health Organization
